# Obesidade, Gordura Corporal e Desfecho Cardiovascular: Além do Índice de Massa Corporal

**DOI:** 10.36660/abc.20210074

**Published:** 2021-05-06

**Affiliations:** 1 Universidade de São Paulo Faculdade de Medicina Hospital das Clinicas São PauloSP Brasil Instituto do Coração do Hospital das Clinicas da Faculdade de Medicina da Universidade de São Paulo, São Paulo, SP – Brasil.

**Keywords:** Obesidade, Tecido Adiposo, Índice de Massa Corporal, Doenças Cardiovasculares, Fatores de Risco, Circunferência Abdominal

A pandemia de obesidade tem estado associada ao aumento de doenças cardiovasculares (DCV). O diagnóstico de DCV é feito com antecedência de dez anos em pessoas obesas.

Embora o índice de massa corporal (IMC) tenha sido amplamente utilizado como o principal índice de obesidade, não é um preditor preciso de doenças cardiovasculares.

Existem outras formas de medir a obesidade, desde uma simples medida da circunferência abdominal (CA) até métodos mais sofisticados, como a impedância bioelétrica e a densitometria por emissão de raios X de dupla energia.

A principal causa da imprecisão do IMC para determinar a distribuição da gordura corporal é que ele pode ser normal em indivíduos com obesidade central determinada pela circunferência abdominal ou com aumento da massa muscular.^1^

Isso gerou o paradoxo da obesidade — pacientes com sobrepeso e obesos com doença cardiovascular apresentam melhor prognóstico do que aqueles com valores normais de IMC.^2^

A discordância entre as duas medidas de obesidade, IMC e CA foi descrita em crianças e jovens brasileiros.^3^ Santos et al.,^3^ constataram que 5,8% dos meninos não obesos, de acordo com o IMC, apresentavam CA acima do valor de corte, enquanto 10,6% dos meninos obesos, de acordo com o IMC, não foram classificados como obesos se a CA fosse usada como critério de classificação.^3^

Em adultos, como mostrado em uma coorte espanhola, no estudo ENRICA,^4^ a prevalência de obesidade central e CA alterada foi mais frequente do que a obesidade por IMC (36% vs. 22,9%); e nos idosos, nos quais, embora a frequência de obesidade por IMC fosse semelhante entre homens e mulheres, a obesidade central mostrou-se cerca de duas vezes mais elevada em mulheres.^5^

Quando os grupos de exposição são diferentes, como em estudos observacionais, é necessário um ajuste estatístico cuidadoso de fatores de confusão para obter estimativas do efeito da exposição livre de viés. Uma simples comparação entre as taxas de incidência pode ser enganosa. Portanto, abordagens computacionais mais sofisticadas foram implementadas.

Quando dois grupos são semelhantes, normalmente calculamos a influência média do grupo, ou seja, a diferença de frequência do desfecho quando algumas características estão presentes ou não. No entanto, se as duas populações estudadas forem diferentes, como em estudos observacionais e epidemiológicos, essa comparação pode levar a resultados enganosos. Assim, por conta dessas características diferentes ou variáveis de confusão, foram desenvolvidas abordagens computacionais por aprendizado de máquina mais sofisticadas.

As variáveis de confusão podem ser analisadas como variáveis mediadoras, ou seja, embora algumas variáveis compartilhem a mesma causa, elas podem influenciar o resultado de forma diferente. Assim, o acúmulo de gordura leva à obesidade central (CA aumentada) e obesidade “geral” (IMC mais elevado) com frequências diferentes. Algumas pessoas podem apresentar obesidade central, mas não apresentar IMC elevado. No entanto, o oposto é incomum. Esta é uma situação em que abordagens computacionais complexas funcionam bem para revelar o efeito de cada situação.

Uma delas é o algoritmo *G-computation*, que se baseia na estimativa do mecanismo de desfecho, da expectativa condicional do desfecho dada a exposição (variável de agrupamento) e covariáveis. Ou seja, qual a probabilidade do desfecho quando presentes a variável de exposição e a de confusão estiver presente. Outro método é o escore de propensão que envolve a estimativa do mecanismo de exposição, ou seja, a probabilidade condicional de ser exposto dado um fator de confusão observado. A probabilidade de associação em uma determinada variável (exposição) quando outra (fator de confusão) está presente.

A ideia subjacente ao pareamento do escore de propensão é que, ao atribuir um escore de propensão a cada indivíduo do estudo, podemos comparar indivíduos em grupos de tratamento diferentes e tentar tornar os indivíduos o mais equivalentes possível para que possamos controlar os fatores de confusão. O resultado diferente seria apenas do tratamento. No entanto, o verdadeiro escore de propensão nunca é realmente conhecido, portanto, sempre há algum nível de incerteza em estudos observacionais.

Normalmente, o escore de propensão (EP) é usado como seu valor inverso denominado peso de propensão inversa. O peso para grupos ativos ou segmentados é 1/EP e para grupos controle, 1/(1-EP).

Outro método que envolve *G-computation* e o escore de propensão é o TMLE (*Targeted Maximum Likelihood Estimation*), que estima tanto a expectativa condicional de um desfecho dada a exposição e variáveis covariáveis (*G-computation*), e a expectativa condicional de exposição sendo determinada por uma variável de confusão.

Nesta edição, Saadati et al.,^6^ utilizam o TMLE para avaliar os efeitos totais (ET) e o efeito direto controlado da influência da obesidade por IMC nos eventos cardiovasculares.

Novamente, encontrou-se uma dissociação entre as medidas de obesidade central e obesidade por IMC.

O resultado final é que as medidas de obesidade central são melhores preditoras de doenças cardiovasculares pelo acúmulo de gordura do que o IMC elevado e são responsáveis por quase todos os riscos de doenças cardiovasculares do acúmulo de gordura corporal.
