# Supplementary material for: Obesidade, Gordura Corporal e Desfecho Cardiovascular: Além do Índice de Massa Corporal
Source: Arq Bras Cardiol. 2021 May 6;116(5):887–8. [Article in Portuguese] doi: 10.36660/abc.20210074 (PMC8121476; doi:10.36660/abc.20210074)
Supplement: Translation

# Translation: Obesity, Body Fat Content and Cardiovascular Outcome: Beyond Body Mass Index

**Affiliations:** 1 São PauloSP Brazil Instituto do Coração do Hospital das Clinicas da Faculdade de Medicina da Universidade de São Paulo, São Paulo, SP – Brazil.

**Keywords:** Obesity, Adipose Tissue, Body Mass Index, Cardiovascular Diseases, Risk Factors, Abdominal Circumference

Obesity pandemic has been associated with an increment of cardiovascular disease (CVD). Diagnosis of CVD is delivered ten years early in obese people.

Although body mass index (BMI) has been widely used as the main index of obesity, it is not an accurate predictor of cardiovascular disease. There are other ways to measure obesity, varying from a simple waist circumference (WC) measurement to more sophisticated methods, like bioelectrical impedance and dual energy X-ray densitometry.

The main cause of BMI inaccuracy to determine body fat distribution is that it may be normal in subjects with central obesity determined by waist circumference or high in increased muscle mass.^1^

This has generated the obesity paradox – overweight and obese patients with cardiovascular disease present better prognosis than those with normal BMI values.^2^

The disagreement between the two measures of obesity, BMI, and WC has been described in Brazilian children and young adults.^3^ Santos et al.^3^ found that 5.8% of non-obese boys, according to BMI, presented WC over the cut-off value, while 10.6% of obese boys, according to BMI, were not classified as obese if the WC was used as a classification criterion.^3^

In adults, as showed in a Spanish cohort, in the ENRICA Study,^4^ the prevalence of central obesity and abnormal WC was more frequent than obesity by BMI (36% vs. 22.9%); and in the elderly, in whom although the frequency of BMI obesity was similar between males and female, central obesity was about twice as high in women.^5^

When the exposure groups are dissimilar, as in observational studies, careful statistical adjustment for confounders is necessary to obtain unbiased estimates of exposure effect. A simple comparison between incidence rates may be misleading, so more sophisticated computational approaches have been implemented.

If two groups are similar, we usually calculate the average group influence, that is, the difference of frequency of outcome when some characteristics are present or not. However, if the two study populations are dissimilar, such as in observational and epidemiological studies, this comparison may give misleading results. Thus, on account of these different or confounding characteristics, more sophisticated computational machine learning approaches have been developed.

The confounding variables may be analyzed as a mediator variable, that is, although some variables share the same cause, they may influence the outcome differently. Thus, fat accumulation leads to central obesity (increased WC) and “general” obesity (higher BMI) with different frequencies. Some people may present central obesity but no elevated BMI. However, the opposite is unusual. This is a situation where complex computational approaches work well in revealing the effect of each one.

One of them is G-computation, which relies on the estimation of the outcome mechanism, the conditional expectation of the outcome given the exposure (grouping variable) and covariates. That is, an exposure variable with other confounding variables is likely to present an outcome. Another method is the propensity score that involves estimation of the exposure mechanism, that is, the conditional probability of being exposed given an observed confounder. The probability of association in a determined variable (exposure) when another (confounder) is present.

The idea underlying propensity score matching is that by giving each individual in the study a propensity score, we can compare individuals in different treatment groups and try to make the individuals as equivalent as possible so that we can control the confounding factors. The different result would be from the treatment only. However, the true propensity score is never really known, so there is always some level of uncertainty in observational studies.

Usually, the propensity score (PS) is used as its inverse value named inverse propensity weight. The weight for active or targeted groups is 1/PS and for control groups, 1/(1-PS).

Another method that involves both G-computation and propensity score is TMLE (Targeted Maximum Likelihood Estimation), which estimates both the conditional expectation of an outcome given the exposure and covariate variables (G-computation), and the conditional expectation of exposure being determined by a confounding variable.

In this issue, Saadati et al.^6^ use TMLE to evaluate the total effects (TE) and the controlled direct effect of BMI obesity influence on cardiovascular events.

Again, a mismatch between central obesity measures to BMI obesity was found.

The final result is that central obesity measures are better predictors of cardiovascular disease from fat accumulation than high BMI, and are responsible for almost all cardiovascular disease risks.
